# P-2083. Validation of the Modified Rapid Test for detection of the Cefazolin Inoculum Effect (CzIE) in bloodstream isolates of Methicillin-*Susceptible Staphylococcus aureus* (MSSA) from North and Latin America

**DOI:** 10.1093/ofid/ofae631.2239

**Published:** 2025-01-29

**Authors:** Lina Paola Carvajal, Sandra Rincon, Diana Panesso, Sara I Gomez Villegas, Kavindra Singh, Karen Jacques-Palaz, Husna Malikzad, Jacob Dziadula, Tanis C Dingle, Susan Butler-Wu, William R Miller, Cesar A Arias, Jinnethe Reyes

**Affiliations:** Universidad El Bosque, Bogotá D.C., Distrito Capital de Bogota, Colombia; Molecular Genetics and Antimicrobial Resistance Unit and International Center of Microbial Genomics, Universidad El Bosque, Bogota, Colombia, Bogota, Distrito Capital de Bogota, Colombia; Houston Methodist Research Institute, Houston, Texas; Brigham and Women's Hospital - Harvard Medical School, Boston, Massachusetts; Houston methodist research institute, Houston, Texas; Houston Methodist Research Institute, Houston, Texas; Houston Methodist Hospital, Houston, Texas; Houston Methodist Research Institute, Houston, Texas; University of Alberta, Alberta Precision Labs, Edmonton, Alberta, Canada; Keck School of Medicine of USC, CA; Houston Methodist Research Institute, Houston, Texas; Houston Methodist and Weill Cornell Medical College, Houston, TX; Molecular Genetics and Antimicrobial Resistance Unit, Universidad El Bosque, Bogota, Distrito Capital de Bogota, Colombia

## Abstract

**Background:**

*S. aureus* is a major human pathogen and a leading cause of bloodstream infections (BSI). Cefazolin is frequently used for severe deep-seated MSSA infections, as in general it displays similar efficacy and a more favorable side effect profile as compared to isoxazolyl-penicillins. In some studies, the CzIE has been associated with therapeutic failures and mortality in MSSA BSI treated with cefazolin, however, current laboratory testing methods are unable to detect the CzIE. The aim of this study was to conduct a blinded evaluation of a modified colorimetric rapid test to detect the CzIE in MSSA isolated from BSI in North and Latin-America.

**Methods:**

99 MSSA recovered from BSI in North America (2019; n=69) and Latin-America (2011-2019; n=30), and previously characterized for the presence of the CzIE (71 CzIE + and 28 CzIE -) using gold-standard broth microdilution at high inoculum (breakpoint >= 16 ug/mL) were included. In a central laboratory in Colombia, a modified rapid colorimetric test (rCT) was performed according to a published protocol with modification that included the use of ampicillin (AMP) discs placed in 1 mL of BHI-broth for induction of β-lactamase expression. The researchers were blinded to the results of the gold standard test to detect CzIE. Diagnostic performance metrics were calculated and typing of BlaZ was performed using whole genome sequence data.

**Results:**

Compared to the gold standard, the modified test showed a sensitivity and specificity of 97% and 86%, respectively, and overall accuracy of 94%. The predominant BlaZ β-lactamase types in the 99 MSSA were type C (70%) and type A (27%). When analyzing the performance of the rCT by BlaZ type, in isolates with type C enzyme the modified rapid test had a sensitivity of 96% and specificity of 82% with an accuracy of 91%. In isolates with BlaZ type A, the sensitivity and specificity of the rCT were 100%.

**Conclusion:**

The modified rapid colorimetric test detected the CzIE in bloodstream MSSA from different geographic areas with a global accuracy of 94%. Notably, the accuracy of the test for BlaZ type A isolates was 100% and for BlaZ type C isolates was 91%. Further, the use of AMP discs proved to be a practical, time-saving modification that did not impair accuracy of the test.

**Disclosures:**

William R. Miller, M.D., Merck: Grant/Research Support|UptoDate: Royalties

